# Spatial and Seasonal Patterns of the Mosquito Community in Central Oklahoma

**DOI:** 10.3390/pathogens11091007

**Published:** 2022-09-03

**Authors:** David Hoekman, Bailee Cummings, Helen Arango, Nicholas Back, Randall Welles, Noah Pelletier, Katelyn Helwig, Christian Escritt, Kayla Thomas, Hailie Fellers, Callie Campbell, Alyssa Wheeler, Raul Iglesias, Hayden Jacobs, Macey Lively, Caio Martinelle B. França

**Affiliations:** 1 Department of Biology, Southern Nazarene University, Bethany, OK 73008, USA; 2 Department of Biology, Redeemer University, Ancaster, ON L9K 1J4, Canada; 3 Department of Biology, Olivet Nazarene University, Bourbonnais, IL 60914, USA; 4 Department of Epidemiology and Biostatistics, School of Public Health, Texas A&M University, College Station, TX 77843, USA

**Keywords:** *Aedes*, CDC light trap, community composition, *Culex*, mosquito, Oklahoma, surveillance, vectors

## Abstract

Mosquitoes (*Culicidae*) are ubiquitous flying insects that function as vectors for several viruses that cause disease in humans. Mosquito abundance and diversity are influenced by landscape features and environmental factors such as temperature and precipitation and vary across seasons and years. The range and phenology of many mosquito species that vector viruses relevant to human health are changing. We sampled mosquito communities in central Oklahoma for four years at thirteen sites, collecting over 25,000 mosquitoes; among these, we identified 27 different species, including several that transmit human pathogens and were collected in suburban backyards. Community composition differed across the landscape and changed from early season to late season and year to year. This effort to describe mosquito communities in Oklahoma is a first step toward assessing and predicting arbovirus risk, an ongoing and dynamic public health challenge.

## 1. Introduction

Vector-borne diseases are a major burden for public health in the United States, and the number of cases of mosquito-borne diseases in humans has increased an order of magnitude in the past 15 years [1]. For example, a range expansion of *Aedes* mosquitoes, specifically *Aedes aegypti* and *Aedes albopictus* from Africa and southeast Asia, respectively, to North America has dramatically changed the landscape for arboviral risk in the United States. [2]. These *Aedes* mosquitoes account for the recently reported intermittent outbreaks and autochthonous transmission of imported Zika, dengue, and chikungunya viruses in the continental United States and associated territories [3]. These events underscore the need to describe patterns of diversity and abundance of mosquito communities in order to understand arboviral risk associated with particular vector species in a region. For example, a West Nile Virus (WNV) outbreak in Oklahoma in 2003 was driven primarily by *Culex tarsalis*, while subsequent outbreaks in 2007 and 2012 were driven by the *Culex quinquefasciatus/pipiens* complex. Because these mosquito species have different habitat and host preferences, the spatial distribution of the WNV outbreaks in Oklahoma varied [4].

Environmental conditions and ecology influence mosquito abundance, species richness, and arboviral risk [5,6,7,8]. Changes in mosquito species dominance driven by changes in environmental conditions can result in different vectors becoming prominent [8,9]. Currently, there are 64 mosquito species that have been reported in Oklahoma [10], distributed across diverse ecoregions of the state in unique assemblages that vary greatly across the landscape [11]. Mosquito population surveillance provides data to support timely and effective control efforts and to reduce the risk of mosquito-borne diseases [12]. In addition, because Oklahoma is in the central flyway for migratory birds, many of which can serve as reservoir hosts for WNV, it is a key location for WNV surveillance efforts [13]. In this study, we describe the mosquito abundance and community composition for four years in four counties that collectively span the Oklahoma City metropolitan area.

## 2. Results

### 2.1. Main Mosquito Species

**Figure 1:**
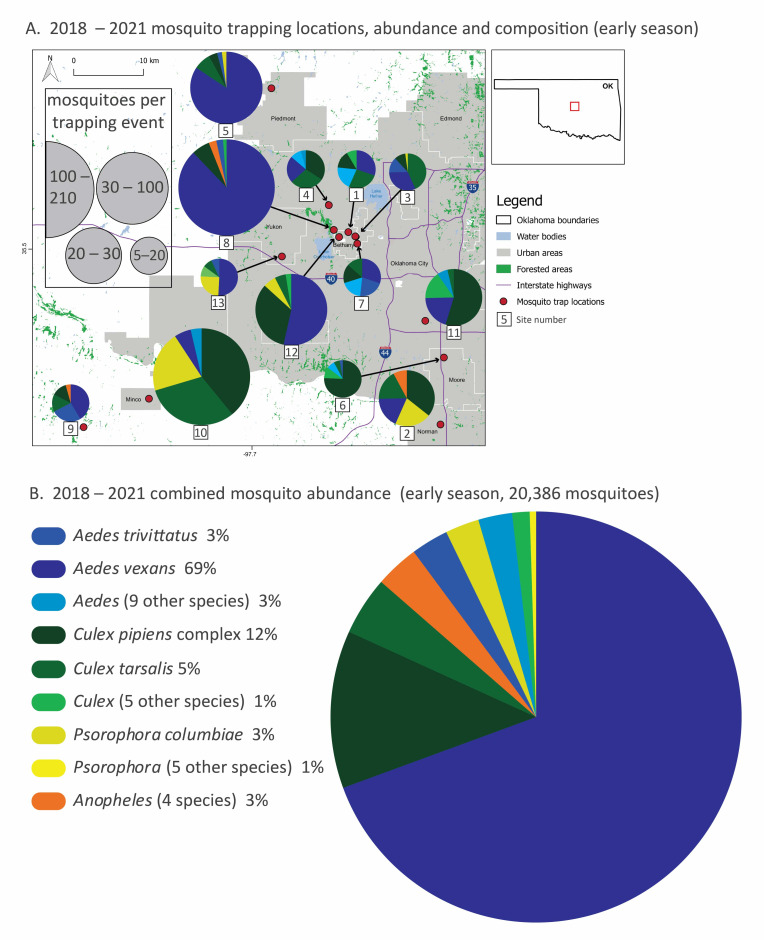

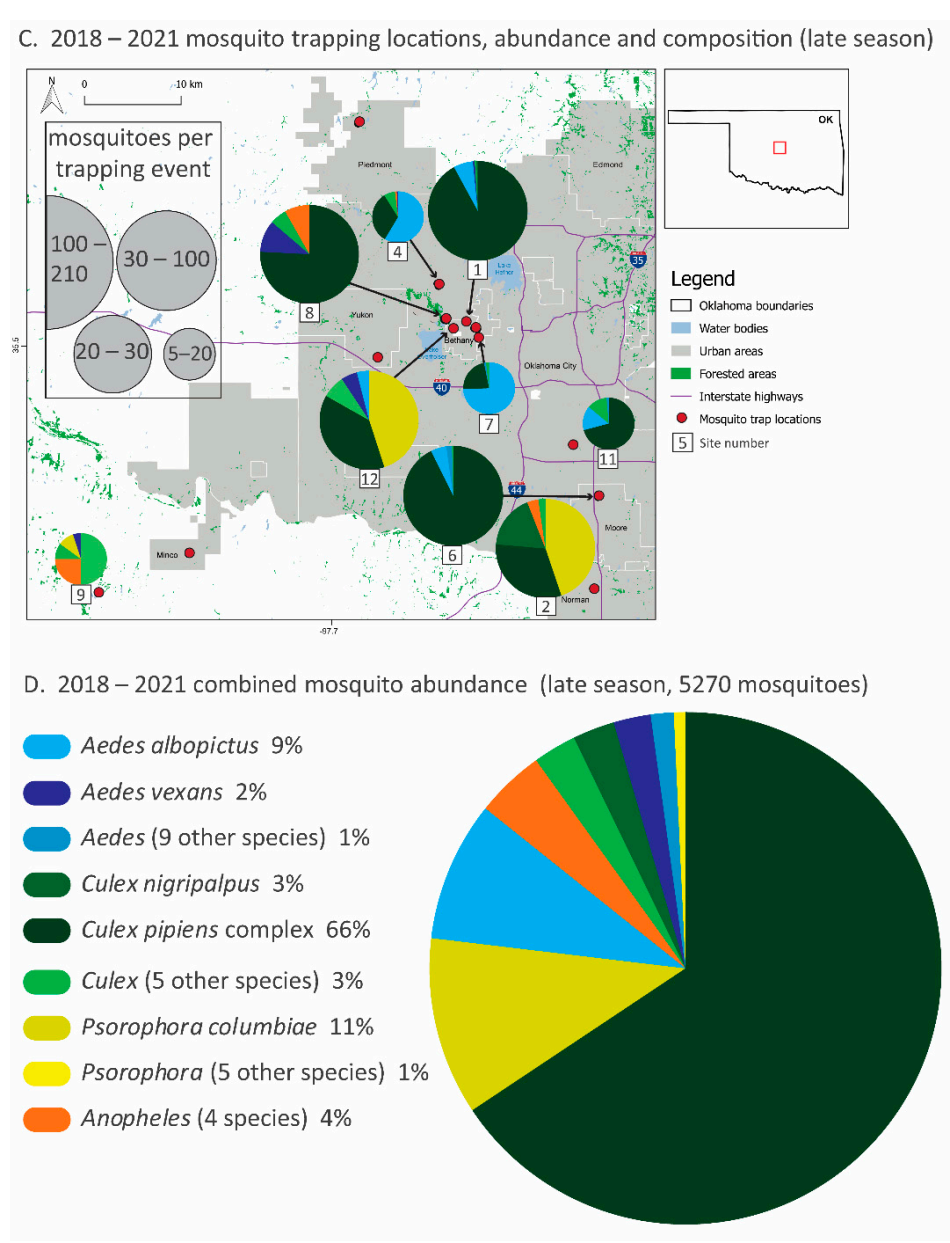
Mosquito species composition, abundance and trapping locations (2018–2021) divided into early (May–June, (**A**,**B**)) and late (July–November, (**C**,**D**)) season sampling. In panels (**A**,**C**), thirteen trapping sites in the greater Oklahoma City metro area, the red box in the Oklahoma state outline, are marked with red dots, and the mosquito relative abundance and species composition of each site are represented with pie graphs. Mosquito collection totals, scaled by collection effort, are represented by the size of the pie graphs and slices represent the proportion of the top-five species at each site. Local municipalities, reservoirs and highways are noted. In panels (**B**,**D**)**,** combined mosquito abundance (total 25,656: about 20,000 from early season sampling and 5000 from late season sampling) from all sites and all four years are displayed by species from the most abundant to the least. Species are color-coded by genus. Species that represent less than 2% of total abundance are pooled at the genus level for clarity. All species totals and percentages are listed in Table 1, and site abundance, species richness and sampling effort are listed in Table 2. A detailed list of sites sampled in this study is provided in the Supplementary Materials (Table S1).

Over the course of four years, 550 trapping events at 13 sites in our four-county Oklahoma sampling area (Table S1) resulted in the collection of 25,656 mosquitoes representing 6 genera and 27 species (Figure 1, Table 1 and Table 2). The mosquito community was dominated by *Aedes* (62%) and *Culex* (30%) species mosquitoes but also included significant numbers of *Psorophora* (5%) and *Anopheles* (3%) species mosquitoes (full species list in Table 1). *Ae. vexans*, a competent vector of West Nile Virus (WNV) and the filarial nematode *Dirofilaria immitis* that causes heartworm disease in dogs [14,15], accounted for 55% of all mosquitoes collected. The second most abundant species was the *Cx. pipiens* complex (23.5%), a known vector of viruses including western equine encephalitis and WNV. *Cx. tarsalis*, another important vector of WNV, contributed an additional 3.7% of mosquitoes in our samples. Mosquito species identification based on COI sequences confirmed the morphological identification for all the mosquitoes of *Aedes* genus and resolved taxonomic conflicts between species of the *Culex* genus, *Cx. pipiens* and *Cx. salinarius*. Similarly, *Coquillettidia pertubans* identification was based on best close match of query in the Barcode of Life data base (BOLD, boldsystems.org).

### 2.2. Temporal Variation in Trapping Success 

The duration of sampling and trapping intensity varied between years (Table 1 and Table 2). Higher sampling intensity, as measured by the number of trapping events, resulted in higher abundance and species richness at the site scale (Table 2). We collected 7804 mosquitoes in 107 trapping events during 15 May 2018–21 June 2018, 3028 mosquitoes in 70 trapping events during 20 May 2019–25 June 2019, 3880 mosquitoes in 149 trapping events during 22 May 2020–22 September 2020 and 10,944 mosquitoes in 224 trapping events during 14 May 2021–16 November 2021.

### 2.3. Early- and Late-Season Mosquito Communities

Mosquito community composition varied spatially and temporally. During early-season sampling (May–June), *Aedes* mosquitoes were most abundant overall and dominated the composition of some sites (e.g., 5 and 7), especially site 8, which had the highest mosquito abundance (Figure 1A,B). *Ae. vexans* alone comprised about 70% of total early season mosquitoes. Early season communities at some sites were composed predominantly of *Culex* species mosquitoes (e.g., 6, 10 and 11), while other sites were more evenly split between species (e.g., 1, 3, 4 and 7). *Psorophora* species mosquitoes, while uncommon overall, were relatively common at some sites (e.g., 2, 10 and 13).

During late-season sampling (July–November), mosquito abundance was lower overall compared to the early season (Figure 1C,D), declining by about 75%. *Culex* species mosquitoes were dominant; *Cx. pipiens* alone comprised over half of the mosquitoes in our late season sampling, dominating some of the highest abundance sites (e.g., 1, 6 and 8). *Cx. nigripalpus* increased in abundance over time, replacing *Cx. tarsalis* in the late season. In contrast, *Aedes* species mosquitoes were far less prominent. When *Aedes* species mosquitoes were abundant at a site (e.g., comprising over half of the mosquitoes caught at sites 4 and 7), it was *Ae. albopictus* rather than *Ae. vexans*. *Psorophora* species mosquitoes were a larger part of the overall mosquito community in the late season, especially at sites 2 and 12. The top five species abundance for each site and season are listed on Table S2.

**Figure 2:**
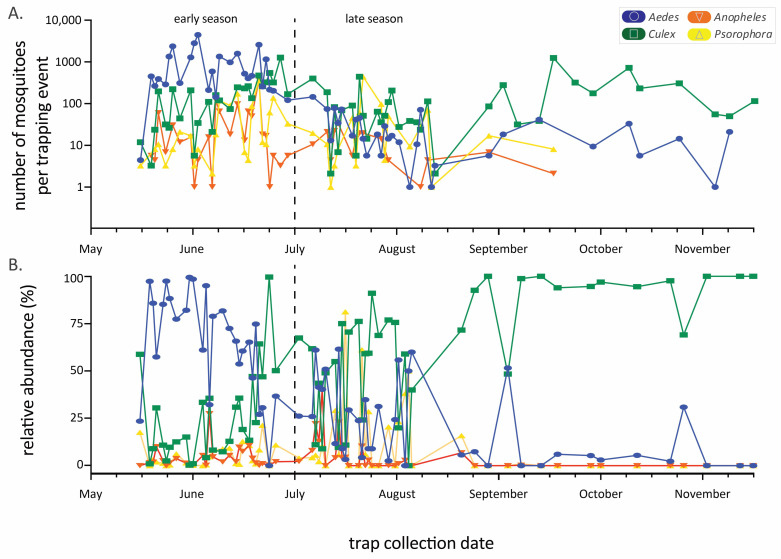
Mosquito abundance by genus throughout the trapping seasons (2018–2021 data combined). Panel (**A**) is the raw number (logarithmic y-axis) of mosquitoes per trapping event while (**B**) is the relative abundance from the same dataset. Date range is from 15 May to 16 November, but the length of the sampling season varied among years. The early/late-season dividing line is drawn at 1 July.

**Figure 3:**
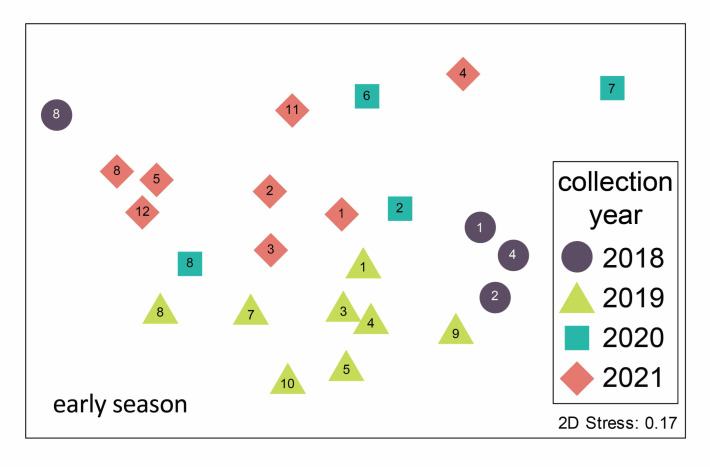
Non-metric multidimensional scaling ordination of mosquito communities trapped in the Oklahoma City metro area in the early season (May–June) 2018–2021. Each point represents the early season mosquito community from a particular site–year combination. Point symbols represent the year of trapping and site numbers are noted (see Figure 1 for locations). Stress is an index of fit between the actual BrayCurtis distance matrix and its representation in two-dimensional ordination space. A stress value of 0.17 is considered low to moderate [16]. The mosquito species assemblages are statistically distinct between years (ANOSIM results in text).

The abundance of the four main genera varied considerably throughout the annual sampling season (Figure 2), generally following a typical seasonal phenology of abundance during warmer months. *Aedes* species mosquitoes were most prevalent early in the season, while *Culex* species predominated later in the season, with a community shift occurring in July (Figure 2B). These seasonal trends in abundance and the predominance of certain genera result in changing community composition through time. Mosquito community composition also differed significantly between sampling years (using early season data, where four years can be compared, one factor ANOSIM global R = 0.47, *p* < 0.001, Figure 3). Specifically, comparing only 2019 and 2020, years in which we used similar numbers of each trap type (Table 1) and trapped at mostly the same sites (Table 2), the mosquito communities were statistically distinct (see Figure 3, pairwise contrast R = 0.61, *p* = 0.002).

## 3. Discussion

Mosquito communities sampled in the Oklahoma City Metropolitan area differed seasonally between years and between sampling locations. These four years of data are part of an ongoing effort to sample mosquito communities through time; associated changes may be an important indicator of environmental change [17]. In another study conducted over a larger spatial extent in Oklahoma, Bradt et al. [11] found variation in urban mosquito communities among three ecoregions within one year. Here, we report within the Central Great Plains ecoregion alone, in central Oklahoma, differences between sites in mosquito community composition across multiple years. However, communities were more similar overall between sites within the same year than between years. As illustrated in Figure 3, although some site numbers do cluster, overall, the symbols (years) group together. This suggests that regional environmental variables, including precipitation and temperature, may be the overriding drivers of community composition [9].

Habitat type has also been recognized as one of the primary drivers of mosquito community composition [18,19,20], and this likely contributed to our finding of variation in mosquito community composition among our sampling sites (Figure 1 and Figure 3). The majority of our sampling sites (all except 5, 8, 9, 10 and 12), were in suburban backyards where mosquitoes were fairly abundant, and some sites contained high species richness (Table 2). For example, we collected 17 species of mosquitoes at site 7, a backyard trapping site, in 2019. However, the sites with highest species richness tended to be rural (e.g., 5, 12), and our single riparian forest site (8) at the Stinchcomb Wildlife refuge alone accounted for half of all mosquitoes collected. The other two rural sites had much lower species richness and abundance due to low sampling effort (only eight trapping events total).

The Stinchcomb Wildlife Refuge (site 8) stands out for several reasons: it is unique among our sampling sites as the only forested site, and it has a unique mosquito community. Although community composition is generally more similar within a year than within a site across years (Figure 3), all four years of sampling at Stinchcomb cluster together in the left side of the ordination plot. The mosquito community composition at Stinchcomb is more similar to other years at Stinchcomb than to other sites during the same year, so the mosquito–environment relationship appears to have a strong influence in this habitat. The site is located along the North Canadian River and is regularly inundated following heavy rain events. It had the highest abundance of mosquitoes and an early season community dominated (90%) by *Ae. vexans,* a floodwater species. Although the composition is similar between years, the dominance of this species was strongly influenced by an extraordinary trapping event in 2018 where we captured about 5000 *Ae. vexans* in a single night in two CDC light traps. This highlights local variability as well as the ability of mosquito populations to grow explosively under the right conditions, in this case, a wet floodplain following heavy rain.

Other sites that contrasted our typical suburban backyard setting resulted in unique mosquito species compositions. *Psorophora* species mosquitoes were rare overall but common at two sites (12 and 2), especially in the late season. Site 12 is classified as rural, although it is situated in a suburban setting. In contrast to suburban sites, it is situated at an equestrian farm, and the local setting is open fields and forest patches. Similarly, site 2, which is in a suburban backyard, borders a large undeveloped area of open fields with forest patches. *Psorophora* species mosquitoes were also more abundant (included in the top five species represented in the pie charts in Figure 1A) in early-season communities at rural sites 10 and 13; both included areas with native grass and patches of forest. Though the role of *Psorophora* species mosquitoes (specifically *Ps. ferox*) in the transmission of WNV is considered to be minor [21], WNV has been detected in all *Psorophora* species collected in this study [22,23].

While previous studies across different regions have identified common species and their seasonality, mosquito communities in Oklahoma are not yet well characterized [10,11,24,25], and this sampling effort is an important contribution toward describing where vector species occur and are common, a critical first step in assessing human arboviral risk. We found several medically and veterinary relevant species (Table 1), often in suburban settings. Seven species of *Aedes* mosquitoes were collected, including *Ae. albopictus,* a known vector of chikungunya, dengue and Zika viruses. *Ae. albopictus* is currently expanding its range in the United States and represents a growing potential for local transmission of these viruses [26,27,28]. In our study, *Ae. albopictus* was an abundant species in several backyard sites (e.g., 1, 4, 7) in densely populated areas, especially in the late season (Figure 1C). *Ae. vexans* was the most abundant species we trapped, dominating early season trapping. Considering its immense numbers and preferential feeding on mammals and birds, it deserves attention as a possible bridge vector for WNV [21]. We also detected *Ae. canadensis*, an important vector of eastern equine encephalitis [29]. Notably, we did not find any *Ae. aegypti,* though previous surveillance has detected this species in southern Oklahoma and northern Texas [24]. The *Cx. pipiens/quinquefasciatus* complex made up 23% of our samples and are the primary known vectors of West Nile Virus in Oklahoma, as indicated by public health virus detection [10] and unpublished data from Oklahoma City County Health Department. 

The sampling effort behind this surveillance study has generated a significant dataset that contributes to our understanding of mosquito communities in suburban settings but is not without limitations. In particular, the sampling design is not well balanced. We collected mosquitoes unevenly at a variety of sites (Table S3), in a number of different landscape contexts, in different years, at different times of year and using different traps. Some sites were sampled every year, while most were not, and sampling effort varied dramatically between sites, even within a year. Trapping site location and frequency was often driven by access and convenience, and our study would be more robust if sites were evenly spread across landscape types (e.g., we only have one forested site) and nearby landscape characteristics (e.g., sources of standing water) were better quantified. The first two years of data only include early-season sampling, predominantly using CDC light traps. The last two years of data offer better coverage of the entire season, but the seasonal shift in mosquito communities is confounded with a shift to gravid traps (Table 1). Ultimately, the mosquito collection effort prioritized collecting blood-fed mosquitoes for virus detection over characterizing how mosquito communities changed across space and time.

Surveillance of mosquito populations is critical for making informed public health decisions about vector control, and this study contributes to our understanding of prevalent vector mosquitoes in the Oklahoma City metropolitan area. Our next steps for this surveillance effort include examining the role of climate and land cover to explain and predict interannual and seasonal changes in mosquito community composition in central Oklahoma.

## 4. Materials and Methods

### 4.1. Field Mosquito Trapping

We selected 13 sites across four counties in central Oklahoma encompassing the Level III Central Great Plains ecoregion, where mixed-grass prairie and riparian woodlands are predominant; all sites are located in the central climate division with similar physiographic and meteorological characteristics [30,31]. The climate in central Oklahoma is subtropical, semi-arid with average annual minimum temperature of 10.5 °C, average annual maximum temperature of 22.2 °C and average annual precipitation of 860 mm [32]. The trapping locations (Figure 1) were selected to represent land-use types within the Oklahoma City metropolitan area, spanning from low- to medium-density urban areas, rural grassland locations and a riparian forest [33,34]. Collection sites were distributed across the range of environmental conditions that characterize this region to obtain a representative sample of the regional mosquito community. Most sites were located in suburban backyards (e.g., mowed grass, landscaping, trees, possible standing water in gutters or birdbaths, Table 2) accessed by permission from property owners. A temporary permit was obtained from the city of Oklahoma City to sample mosquitoes at the Stinchcomb Wildlife Refuge (site 8), which includes a bottomland hardwood forest. Rural locations were primarily pastures dominated grasses and shrubs. Sites at which sampling was conducted varied between years (Table 2), with all but one site (#10) sampled for at least two years. Weekly adult mosquito trapping took place between May and June in four years (2018–2021); the sampling season was extended into September in 2020 and November in 2021. Following Bradt et al. [11] we defined sampling during May–June as “early season” and sampling during and after July as “late season.”

Sampling was conducted with two types of mosquito traps to capture a diverse mosquito community. We used CDC miniature light traps (Bioquip, Rancho Dominguez, CA, USA), baited overnight with dry ice as a CO_2_ source, because of their superior effectiveness in terms of catch abundance [11]. In addition, beginning in 2019, we also sampled using CDC gravid traps (John W. Hock Company, Gainesville, FL, USA) baited with water infused with locally harvested grass for 48 hours. Sampling at each site involved six CDC light traps and two gravid traps in 2019, eight light traps and six gravid traps in 2020, and eight traps of each type in 2021. The number of sampling events per site per year are listed in Table 2. Upon collection, we transported catch bags containing mosquitoes from the field in coolers and stored them at −20 °C until processing (usually on the same day).

### 4.2. Lab Processing

Mosquitoes were sorted and identified based on morphology [35]. In brief, mosquitoes were euthanized by freezing at −20 °C, sorted, and adult female specimens identified to species using morphological keys from Darsie and Ward [36], Burkett-Cadena [37], and Walter Reed Biosystematics Unit (http://www.wrbu.org/VecID_MQ.html (accessed on 15 June 2018)). Adult female specimens were pooled by trap and species (pools size 5–35 individuals), placed in screw cap tubes and stored at −80 °C.

### 4.3. Sanger Sequencing

To resolve taxonomic uncertainty, we used genetic analysis to confirm morphological species identifications. Genomic DNA was extracted from selected individual adult female specimens using the Quick-DNA^TM^ Tissue/insect kit (Zymo Research, Irvine, CA, USA). The mitochondrial cytochrome c oxidase subunit I (COI) gene was amplified using Folmer primers [38], and bead-cleaned (KAPA Biosystems, Wilmington, MA, USA) fragments were sequenced using ThermoFisher’s BigDye Terminator v3.1 Sequencing Kit per standard protocols. Reactions were cleaned using Sephadex columns and loaded onto the 4-capillary SeqStudio Genetic Analyzer from Applied Biosystems (ThermoFisher, Waltham, MA, USA). Raw sequences were visualized, trimmed and corrected using Geneious Prime v2022.0.1 [39] entered into the Barcode of Life Data system and matched to previously identified specimens in the BOLD database (https://www.boldsystems.org/ (accessed on 5 May 2020)). COI sequences were deposited to Genbank available under the accession numbers OL437322-OL437326.

### 4.4. Mosquito Community Assemblage Analysis

We examined differences in mosquito community composition between seasons and years using multivariate ordination analyses and randomization tests. Samples collected within a given year were divided into early (May–June) and late (July–November) season site-level communities. Mosquito abundance was square-root transformed, and a Bray–Curtis distance matrix was calculated for the taxa–site matrix to measure the similarity of mosquito community composition between sites. First, a nonmetric multidimensional scaling (NMS) ordination was performed to visualize similarities between sites (PRIMER-E Version 6.1.5; PRIMER-E Ltd., Plymouth, UK). In an NMS ordination, the mosquito community at a site is represented by a point, and points that are closer together are more similar in terms of the relative abundance of species present at that site, whereas points that are further apart have less similar mosquito communities. Subsequently, we tested the effect of the year and season on mosquito community composition using the Bray–Curtis distances and associated analysis of similarity (ANOSIM) tests [16]. Site was excluded as a factor because of low replication and large variation in within-site sampling effort. Because early- and late-season mosquito communities differed in terms of community composition, and sampling effort varied considerably between seasons (notably, lack of late season sampling during 2018–2019), we repeated the above analysis using only early season data to test whether mosquito community composition changed across years.

## Figures and Tables

**Table 1:** Mosquito catch numbers by species collected in the Oklahoma City metro area in 2018–2021. The table summarizes the abundance for each species by year and early/late season, as well as their percentage of total abundance by species. In addition, for each year/season combination, the number of sites sampled, number of trapping events, ratio of trap types and sampling date ranges are listed.

	2018	2019	2020	2020	2021	2021	
**Sampling season**	Early	Early	Early	Late	Early	Late	
**No. sampling sites**	9	11	9	8	8	4	
**Total trapping events**	107	70	62	87	135	100	
**Light/Gravid events**	107/0	57/13	26/36	25/62	78/57	0/69	
**Sampling period**	15 May –21 June	May 20 –June 25	22 May –30 June	1 July– 22 September	14 May –30 June	1 July– 16 November	
**Species**							**% Total**
*Aedes albopictus*	79	113	57	343	40	128	3.11
*Aedes atropalpus*	-	1	-		1	1	0.01
*Aedes canadensis*	-	-	1	13	30	-	0.17
*Aedes epacticus*	4	2	13	39	43	0	0.39
*Aedes sollicitans*	24	52	0	7	6	8	0.33
*Aedes triseriatus*	13	17	11	4	39	5	0.36
*Aedes trivittatus*	-	440	8	4	167	2	2.35
*Aedes vexans*	7272	1184	284	112	5333	15	55.16
***Aedes* Total**	**7395**	**1819**	**374**	**522**	**5659**	**159**	
*Culex coronator*	5	-	1	13	3	-	0.09
*Culex erraticus*	2	-	23	42	2	-	0.27
*Culex nigripalpus*	20	4	7	132	52	9	0.86
*Culex pipiens*	325	460	257	1388	1478	2135	23.55
*Culex restuans*	-	-	0	8	46	2	0.22
*Culex salinarius*	-	-	2	68	276	15	1.41
*Culex tarsalis*	39	674	14	8	316	10	3.71
***Culex* Total**	**391**	**1169**	**304**	**1659**	**2173**	**2171**	
*Anopheles crucians*	-	-	74	30	286	5	1.54
*Anopheles pseudopunctipennis*	19	23	106	29	2	2	0.69
*Anopheles punctipennis*	-	36	11	14	35	4	0.37
*Anopheles quadrimaculatus*	18	6	38	57	55	2	0.67
***Anopheles* Total**	**37**	**65**	**229**	**130**	**378**	**13**	
*Psorophora ciliata*	-	4	0	3	3	1	0.04
*Psorophora columbiae*	21	163	34	574	327	24	4.43
*Psorophora cyanescens*	-	-	2	32	54	-	0.34
*Psorophora ferox*	3	9	1	-	16	2	0.15
*Psorophora howardii*	-	-	1	1	-	-	0.01
***Psorophora* Total**	**24**	**176**	**38**	**610**	**400**	**27**	
*Orthopodomyia signifera*	-	3	-	1	-	-	0.02
*Coquillettidia perturbans*	-	-	-	11	-	-	0.04
Unknown	4	16	5	-	-	-	0.05
**Grand Total** **Mosquitoes**	**7804**	**3028**	**945**	**2935**	**8609**	**2335**	**25,656**

**Table 2:** The number of unique species (richness) of mosquitoes by site, year and trapping season and mosquitoes collected (abundance) and trapping events by site and trapping season. Early trapping season (yellow shading) is defined as trapping in May and June, while late (blue shading) is any trapping later in the year (mostly July and August). In the first two years of the study, only early sampling was conducted, while late season sampling was added in the last two years. The site numbers correspond to the sites in Figure 1. Most sites are suburban backyards, while a few are more rural, and one site is a riparian forest in a wildlife refuge.

		Species Richness									Abundance			Trapping Events		
**Site** **Number**	**Landscape Setting**	**2018** **Early**	**2019** **Early**	**2020** **Early**	**2020** **Late**	**2021** **Early**	**2021** **Late**	**Early** **Total**	**Late** **Total**	**Site** **Total**	**Early**	**Late**	**Total**	**Early**	**Late**	**Total**
1	Suburban backyard	12	11			12	7	19	7	19	475	1566	2041	43	18	61
2	Suburban backyard	9		9	12	13		15	12	17	613	552	1165	29	8	37
3	Suburban backyard		8	2				9		9	191		191	10		10
4	Suburban backyard	8	7	3	6	7	8	13	11	18	279	279	558	34	15	49
5	Rural	4	7	1		19		20		20	2576		2576	29		29
6	Suburban backyard		4	11	9			11	9	14	246	769	1015	19	10	29
7	Suburban backyard		17	5	9			17	9	21	711	160	871	48	14	62
8	Riparian forest	14	12	17	15	19	13	23	18	24	11,818	859	12,677	57	24	81
9	Rural		7		5			7	5	10	49	20	69	3	2	5
10	Rural		11					11		11	379		379	3		3
11	Suburban backyard				7	10		10	7	12	474	153	627	16	18	34
12	Rural			9	23	19	12	20	26	27	2225	912	3137	29	19	48
13	Suburban backyard			3		16		17		17	350		350	20		20

## Data Availability

The data used for the community assemblage presented in this study are openly available in FigShare at 10.6084/m9.figshare.20216960.

## Supplementary Materials

The following supporting information can be downloaded at: https://www.mdpi.com/article/10.3390/pathogens11091007/s1, Table S1. List of sample sites in the Oklahoma City metro area. The numbers correspond to the twelve sites in Figure 1. The site names are generally a household or municipality name. The landscape setting categories are suburban backyard, rural and riparian forest. Latitude and longitude are reported in decimal degrees. Table S2. Top five most abundant species per each site and their seasonal totals for the years sampled. Table S3. Because trapping effort varied among sites and which sites were sampled varied among years, we have summarized trapping effort (trap nights) and total abundance here by site and by trap type, namely CDC light trap (light) and CDC gravid traps (Gravid).
