# Supplementary material for: Spatial and Seasonal Patterns of the Mosquito Community in Central Oklahoma
Source: Pathogens. 2022 Sep 3;11(9):1007. doi: 10.3390/pathogens11091007 (PMC9502914; doi:10.3390/pathogens11091007)
Supplement: Supplementary file 1 [file pathogens-11-01007-s001.zip › pathogens-1827550-supplementary.pdf]

Table S1. List of sample sites in the Oklahoma City metro area. The numbers correspond to the sites in Figure 1. The site names are generally a household or municipality name. The landscape setting categories are suburban backyard, rural and riparian forest. Latitude and longitude are reported in decimal degrees.

Site number	Site name	County	Landscape setting	Latitude	Longitude
1	N SNU	Oklahoma	Suburban backyard	35.515428	-97.628656
2	NW Norman	Cleveland	Suburban backyard	35.273148	-97.494682
3	Rockwell	Oklahoma	Suburban backyard	35.519856	-97.640278
4	Northwest OKC	Oklahoma	Suburban backyard	35.555492	-97.671285
5	Piedmont	Canadian	Rural	35.704071	-97.762140
6	Moore	Oklahoma	Suburban backyard	35.358691	-97.469058
7	Southeast Bethany	Oklahoma	Suburban backyard	35.506219	-97.626057
8	Stinchcomb	Oklahoma	Riparian forest	35.522475	-97.663681
9	Minco 1	Grady	Rural	35.305378	-98.058573
10	Minco 2	Grady	Rural	35.270945	-98.058573
11	Southwest OKC	Oklahoma	Suburban backyard	35.370093	-97.525147
12	Equestrian	Oklahoma	Rural	35.518641	-97.649372
13	West OKC	Oklahoma	Suburban backyard	35.441111	-97.687222

Table S2. List of only the top five most abundant species for all sample sites in the Oklahoma City metro area. The site number correspond to the sites in Figure 1. The number of species reported are the collection totals resulting from all trapping efforts for all years sampled at that site, separated by early and late season when applicable. The data represented here is a detailed list of the graphic representation of Figure 1.

Site number	Site name	Top five species collected	
		Early Season	Late Season
**1**	N SNU	110 *Ae. vexans*	436 *Cx. pipiens*
		96 *Cx. tarsalis*	32 *Ae. albopictus*
		73 *Ae. albopictus*	28 *Cx. quinquefasciatus*
		31 *Cx. nigripalpus*	3 *Ae. vexans*
		29 *Cx. quinquefasciatus*	3 Cx. salinarius
**2**	NW Norman	161 *Cx. quinquefasciatus*	233 *Ps. columbiae*
		112 *Ps. columbiae*	117 *Cx. quinquefasciatus*
		96 Ae. *vexans*	92 *Cx. nigripalpus*

		93	*Cx. tarsalis*	47	*Cx. pipiens*
		42	*An. crucians*	19	*An. quadrimaculatus*
**3**	Rockwell	80	*Cx. tarsalis*		
		58	*Ae. vexans*		
		25	*Ae. trivittatus*		
		17	*Cx. pipiens*		
		4	*Ps. columbiae*		
**4**	Northwest OKC	70	*Cx. tarsalis*	157	*Ae. albopictus*
		65	*Cx. pipiens*	70	*Cx. pipiens*
		55	*Ae. vexans*	19	*Cx. erraticus*
		23	*Ae. albopictus*	17	*Cx. quinquefasciatus*
		17	*Cx quinquefasciatus*	3	*An. punctipennis*
**5**	Piedmont	2075	*Ae. vexans*		
		189	*Cx. tarsalis*		
		60	*Cx. pipiens*		
		53	*Ae. trivittatus*		
		49	*Ps. columbiae*		
**6**	Moore	101	*Cx. pipiens*	644	*Cx. pipiens*
		73	*Cx. quinquefasciatus*	65	*Cx. quinquefasciatus*
		22	*Cx. erraticus*	39	*Ae. albopictus*
		16	*Ae. albopictus*	9	*Ae. epactius*
		12	*Cx. tarsalis*	5	*Ae. canadensis*
**7**	Southeast Bethany	193	*Ae. vexans*	117	*Ae. albopictus*
		138	*Ae. trivittatus*	32	*Cx. pipiens*

		118	*Ae. albopictus*	3	*Cx. quinquefasciatus*
		101	*Cx. pipiens*	2	*Ae. epactius*
		87	*Cx. tarsalis*	2	*Cx. coronator*
**8**	Stinchcomb	10040	*Ae. vexans*	78	*Ae. vexans*
		386	*Cx. quinquefasciatus*	41	*Cx. quinquefasciatus*
		310	*Cx. pipiens*	31	*An. quadrimaculatus*
		294	*An. crucians*	30	*An. crucians*
		260	*Ae. trivittatus*	26	*An. pseudopunctipennis*
**9**	Minco 1	19	*Ae. vexans*	10	*Cx. nigripalpus*
		12	*Ae. trivittatus*	5	*An. punctipennis*
		7	*Cx. tarsalis*	2	*Cx. quinquefasciatus*
		6	*Cx. pipiens*	2	*Ps. columbiae*
		2	*An. pseudopunctipennis*	1	*Ae. vexans*
**10**	Minco 2	140	*Cx. pipiens*		
		112	*Cx. tarsalis*		
		73	*Ps. columbiae*		
		20	*Ae. vexans*		
		13	*Ae. sollicitans*		
**11**	Southwest OKC	232	*Cx. quinquefasciatus*	59	*Cx. quinquefasciatus*
		89	*Ae. vexans*	48	*Cx. pipiens*
		70	*Cx. salinarius*	23	*Ae. albopictus*
		27	*Ae. canadensis*	16	*Cx. erraticus*
		16	*Cx. tarsalis*	3	*Cx. coronator*
**12**	Equestrian	1112	*Ae. vexans*	349	*Ps. columbiae*

		526	*Cx. pipiens*	296	*Cx. pipiens*
		166	*Cx. quinquefasciatus*	58	*Cx. salinarius*
		120	*Ps. columbiae*	41	*Ae. vexans*
		104	*Cx. tarsalis*	31	*Ae. albopictus*
**13**	West OKC	153	*Ae. vexans*		
		75	*Ps. columbiae*		
		28	*Cx. restuans*		
		24	*Cx. tarsalis*		
		21	*Ae. trivittatus*		

Table S3. Because trapping effort varied among sites and which sites were sampled varied among years, we have summarized trapping effort (trap nights) and total abundance here by site and by trap type, namely CDC light trap (light) and CDC gravid traps (Gravid). For each site and trap type, the first number is the trapping effort (number of trap nights) and the second number is the total abundance. For example, at site 1 in 2018 we collected 142 mosquitoes during 51 trap nights, all using CDC light traps.

Site Number	Trap Type	2018 Early	2019 Early	2020 Early	2020 Late	2021 Early	2021 Late
1	Light	51/142	9/141	N/A	N/A	12/154	
	Gravid		3/26			3/46	32/1541
2	Light	11/45	1/4	5/123	8/452	12/471	N/A
	Gravid						
3	Light	N/A	8/188	N/A	N/A	N/A	N/A
	Gravid						
4	Light	20/55	3/149		1/2		
	Gravid			4/10	11/234	8/65	4/59
5	Light	10/27	3/362		N/A	13/2173	N/A
	Gravid		2/42	1/1		11/78	
6	Light	N/A	2/49	1/6		N/A	N/A
	Gravid			15/229	11/770		
7	Light	N/A	14/575	6/7	2/12	N/A	N/A
	Gravid		7/99	4/34	12/148		
8	Light	15/7535	10/965	7/362	7/218	12/2157	
	Gravid		1/0	7/140		9/353	27/651
9	Light	N/A	1/49	N/A	N/A	N/A	N/A
	Gravid						
10	Light	N/A	2/379	4/10	1/3	N/A	N/A
	Gravid				2/7		
11	Light	N/A	N/A			3/44	N/A
	Gravid				14/342	15/497	
12	Light	N/A	N/A	3/7	6/382	8/1480	
	Gravid			4/14	12/365	11/728	4/61
13	Light	N/A	N/A		N/A	18/363	N/A
	Gravid			2/2			
Total events/abundance	Light	107/7804	53/2861	26/515	25/1169	78/6842	
	Gravid		13/167	36/430	62/1766	57/1767	69/2335
